# Sporulation environment drives phenotypic variation in the pathogen *Aspergillus fumigatus*

**DOI:** 10.1093/g3journal/jkab208

**Published:** 2021-06-17

**Authors:** S Earl Kang, Brandi N Celia, Douda Bensasson, Michelle Momany

**Affiliations:** Fungal Biology Group & Plant Biology Department, University of Georgia, Athens, GA 30602, USA; Fungal Biology Group & Plant Biology Department, University of Georgia, Athens, GA 30602, USA; Institute of Bioinformatics & Plant Biology Department, University of Georgia, Athens, GA 30602, USA; Fungal Biology Group & Plant Biology Department, University of Georgia, Athens, GA 30602, USA

**Keywords:** sporulation, conidia, heterogenetity, flow cytometry

## Abstract

*Aspergillus fumigatus* causes more than 300,000 life-threatening infections annually and is widespread across varied environments with a single colony producing thousands of conidia, genetically identical dormant spores. Conidia are easily wind-dispersed to new environments where they can germinate and, if inhaled by susceptible hosts, cause disease. Using high-throughput single-cell analysis via flow cytometry we analyzed conidia produced and germinated in nine environmentally and medically relevant conditions (complete medium, minimal medium, high temperature, excess copper, excess iron, limited iron, excess salt, excess reactive oxygen species, and limited zinc). We found that germination phenotypes vary among genetically identical individuals, that the environment of spore production determines the size of spores and the degree of germination heterogeneity, and that the environment of spore production impacts virulence in a *Galleria mellonella* host.

## Introduction

Fungal diseases kill over 1.5 million people each year (Brown *et al.* 2012; Bongomin *et al.* 2017). Rather than spreading patient-to-patient, fungal diseases are acquired from the environment or host normal flora. Nine of the ten most common agents of fungal disease can be spread via spores (Botts and Hull 2010; Brown *et al.* 2012). Breaking dormancy, or germinating, is arguably the most important step in pathogenesis for these fungi. Historically studies have focused on the germination environment, addressing factors such as temperature, inoculum density, carbon source, nitrogen source, and pH (Brown 1922; Loo 1976; Osherov and May 2001; Araujo and Rodrigues 2004; Wang *et al.* 2019). However, despite the wide range of environments in which fungal spores are produced and their importance as disease agents, the impact of sporulation environment on germination has been largely ignored. To determine whether the environment of sporulation influences germination, we performed sporulation/germination swap experiments in which spores of the fungal pathogen *Aspergillus fumigatus* were produced in one of nine environmentally or medically relevant conditions and then shifted to one of the same nine conditions for germination. By monitoring hundreds of thousands of spores, we found that germination phenotypes vary among genetically identical individuals and that the environment of spore production determines the size of spores and the degree of germination heterogeneity. By testing the ability of spores produced in different environments to kill a *G. mellonella* host, we also found that sporulation environment impacts virulence.

## Materials and methods

### Fungal strains, cultivation, and preparation of conidia

*A. fumigatus* CEA10 was cultivated on 1.5% agar solid complete medium (CM) or minimal medium (MM) as previously described (Momany *et al.* 1999) with modifications as described in Table 1. For conidial stock preparation, conidia were produced on complete media, harvested in sterile water, and 1 × 10^6^ conidia in 500 µl of ddH2O were plated in a homogenous layer on 25 ml of solid 1% glucose *Aspergillus* MM with modifications described in Table 1 in 90 mm plates in 3 technical replicates. Plates were incubated in the dark, stored upside down at 37°C or 50°C for 72 hours. *A. fumigatus* conidia from 3 plates were harvested by overlaying plates with 25 ml sterile ddH2O, combining conidia and filtering through 22–25 µm Miracloth (MilliporeSigma, St. Louis, MO, USA). Conidia were washed twice in ddH2O and counted using a hemocytometer.

**Table 1:** Sporulation and germination conditions

Abbreviation	Description	Medium	Temperature (°C)
CM	Complete medium	Nutrient-rich undefined medium containing yeast extract, glucose, nitrogen, and vitamins	37
MM	Minimal medium	Nutrient-rich defined synthetic medium containing glucose, nitrogen, and vitamins	37
50**°**C	High temperature stress	MM	50
+Cu	Copper stress	MM with 1 mM CuSO_4_	37
+Fe	Excessive iron stress	MM with 10 mM FeSO_4_	37
–Fe	Iron limiting stress	MM without FeSO_4_	37
NaCl	Osmotic or salt stress	MM with 0.5 M NaCl	37
H_2_O_2_	Reactive oxygen species (ROS) stress	MM with 2 mM H_2_O_2_	37
–Zn	Zinc limiting stress	MM without ZnSO_4_	37

### Germination assay

Conidia from 3 plates were pooled and identical aliquots of 3–5 × 10^5^ C/ml were added to liquid germination conditions described in Table 1 (Araujo and Rodrigues 2004). Cultures were incubated for 6 hours at 37°C or 50°C @ 250 rpm in dark, then fixed with 2.5% formaldehyde. Eighty-one conditions were analyzed in total. Controls included conidia fixed at 0 hour in liquid germination conditions.

### Analysis of germination/flow cytometry

Flow cytometry was performed at the Center for Tropical and Emerging Global Diseases Cytometry Shared Resource Laboratory at the University of Georgia on a CyAn ADP using Summit, version 4.3 (Beckman Coulter, Fullerton, CA, USA). Between 20,000 and 250,000 events (cells) were analyzed in four replicates for each fixed pre- and post-germination sample. Due to the sensitivity of flow cytometry and small particulates in the germinated samples, forward scatter and side scatter values smaller than fixed ungerminated conidia were filtered from the analysis. FlowJo flow cytometry analysis software, version 10 (Tree Star, Ashland, OR, USA) was used for analysis and histogram. Histogram represents the linear scaled forward scatter data to better visualize the variation in germination. Conidial morphologies (including the absence of significant clumping) were verified by microscopic observation for all experiments and by Amnis ImageStream (Amnis MerckMillipore Sigma, Seattle, WA, USA) for three experiments.

### Statistical analysis of germination

Forward scatter scaled linear or log data were combined for each condition from all replicates. Linear and log data were checked for normality using D’Agostino-Pearson test (D’Agostino *et al.* 1990). Due to nonparametric distribution, comparison between multiple groups were analyzed by Kruskal–Wallis test followed by one-sided Dunn’s multiple comparison test(Dunn 1964) using GraphPad Prism version 8 (GraphPad Software, La Jolla, CA, USA). Robust coefficient of variance (rCV) was calculated using 100 * 1/2 (Intensity [at 84.13 percentile]—Intensity [at 15.87 percentile])/Median using FlowJo v10 (Tree Star, Ashland, OR, USA). Pearson correlation analysis followed by a two-tailed test was performed to assess the relationship between median log forward scatter (growth) and rCV (variation) in a given germination condition using GraphPad Prism version 8 (GraphPad Software, La Jolla, CA, USA).

### Viability assay—live/dead staining

For viability assays, two replicates of unfixed cells (conidia and germlings) were co-stained with 10 µg/ml fluorescein diacetate (FDA) and 2 µg/ml propidium iodine (PI) for 5 minutes in the dark, then 20,000 events were analyzed immediately using flow cytometry to measure size (forward scatter) and fluorescence. Controls included unstained and FDA, PI, and FDA+PI stained live and dead (ethanol-killed) cells.

### *Galleria mellonella* infections

*G. mellonella* larvae (waxworms.net, St. Marys, OH, USA) were stored in wood shavings in the dark at room temperature prior to use. Wax-moths in the final instar larval stage were used for injections using the method of Jackson et al (Jackson *et al.* 2009). Briefly *G. mellonella* was placed at 20°C for 25 minutes and placed on ice prior to injections. Using a cotton swab dipped in ethanol, *G. mellonella* prolegs were cleaned and the left leg of the 3rd set of prolegs via the hemocoel were injected with a total of 10^6^ conidia in 5 µl of PBS solution using a Hamilton syringe. Fifteen larvae were infected for each condition. After injections, larvae were incubated in petri plates at 37°C for 72 hours with observation every 12 hours. *G. mellonella* were considered dead if they had no response to touch. *G. mellonella* larvae were shipped to the lab in separate batches. Five replicate experiments were performed. No injection and PBS only controls were included for all replicates. *G. mellonella* was infected with conidia that had been produced under five different conditions: MM, 50°C, +Fe, NaCl, and –Zn. Fifteen larvae were injected with each conidium type in 5 separate replicates, so in total 375 larvae were injected.

### Statistical analysis of infection assays

To test for differences among the batches of larvae and between the 5 sporulation conditions on host survival, we fit a generalized linear mixed-effects model (GLMM) using R (version 4.0.2) and the glmer function from the lme4 package (version 1.1-26; Bates *et al.* 2020). For this survival analysis, the time last seen alive in hours (Time) was set as the response variable. We used the gamma family of errors because variance increases with increasing mean host age at death (Crawley 2012)*.* Replicates were included in the model as a random effect, and we tested for differences among conidia exposed to 5 different sporulation Treatments: MM, 50 C, Fe, NaCl and Zn; Time ∼ Treatments + (1| Replicates). The model was fit with default settings for glmer; with maximum likelihood using the Laplace approximation. We tested for differences among treatments through a likelihood ratio test on nested models using the drop1 function with the “Chisq” option, and *P*-values in Table 3 are based on the default asymptotic Wald tests for glmer models (Bates *et al.* 2020). As a control, we repeated the above analysis using a mixed-effects Cox model implemented in R with coxme (version 2.2-16). This second analysis confirms the significant differences among replicates (Supplementary Figure S3; Likelihood ratio test, *df* = 1, *P* = $2\times 10^{-10}$) and among treatments of conidia (Table 3; Likelihood ratio test, *df* = 4, *P* = 0.003). The data table and R script used for analyses are available at https://github.com/bensassonlab/data/tree/master/kang_etal20.

**Table 3:** *G. mellonella* estimated survival times (in hours) after injection with *A. fumigatus* conidia produced in differing sporulation conditions

	50°C	+Fe	MM	NaCl	−Zn
Replicate 1 (*N* = 15)	24	33	35	36	35
Replicate 2 (*N* = 15)	20	25	26	26	26
Replicate 3 (*N* = 15)	17	21	22	23	23
Replicate 4 (*N* = 15)	25	34	35	37	36
Replicate 5 (*N* = 15)	26	35	37	38	39
Average (*N* = 75)	22	30*	31**	32***	32***

Averages were calculated over 5 experimental replicates, and *T*-tests for the difference between conidia sporulated at 50°C and other treatments are from a generalized linear mixed model (see *Materials and Methods*): *P < 0.005, **P < 0.001, ***P < 0.00005.

### Data availability

Strains and plasmids are available upon request. The authors affirm that all data necessary for confirming the conclusions of the article are present within the article, figures, and tables. Supplemental material available at *G3* online. 

## Results and discussion

We hypothesized that exposure to specific stresses during sporulation might lead to better germination in the same or related conditions. To test this hypothesis, we performed single-cell experiments in which *A. fumigatus* was sporulated under nine environmentally and medically relevant conditions (Errasquin *et al.* 2002; Tepsic *et al.* 2006; Haas 2012; Amich and Calera 2014) and the resulting conidia were transferred to all nine conditions for germination (Table 1). To avoid induction or selection of mutations during sporulation, we did not use serial passaging; rather, identical aliquots of inoculum were incubated for 72 hours on nine types of solid medium for the production of conidia, and identical aliquots of conidia from each condition were transferred directly to nine types of liquid medium for germination (Supplementary Figure S1).

**Figure 1:**
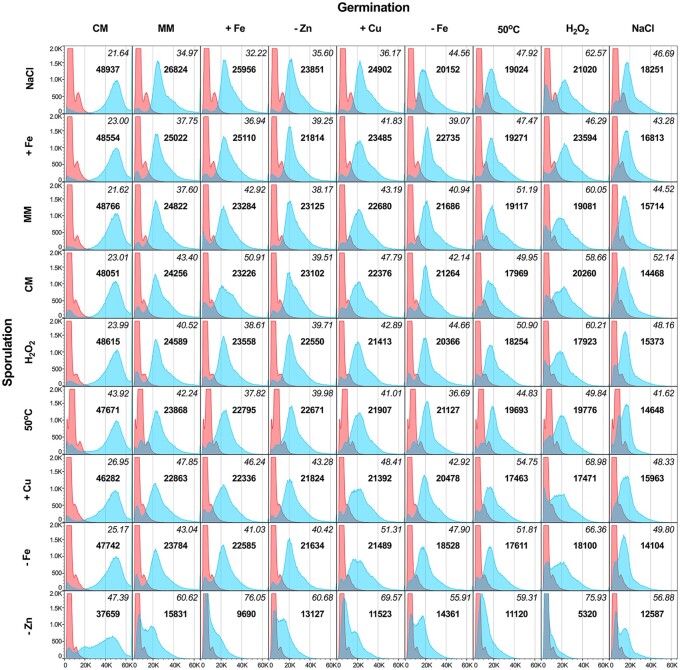
Sporulation conditions impact germination of *A. fumigatus* conidia. Conidia produced in one of nine sporulation environments were transferred to all nine conditions for germination. The *x*-axis shows the linear forward scatter, an indication of relative size. The *y*-axis shows the number of events (cells) counted. Red peaks show forward scatter of dormant conidia from each sporulation condition measured before germination. Blue peaks show forward scatter after 6 hours incubation in each germination condition. Bold values are the median of linear forward scatter values after germination. Italicized values are the robust coefficient of variation (normalized standard deviation around the median) of linear forward scatter values after germination.

After 6 hours incubation we used flow cytometry to detect any increase in cell size, a clear indication that germination had been initiated. The entire 9 by 9 sporulation/germination swap experiment was repeated four times. We recorded forward scatter for approximately 20,000 conidia and germlings for each condition in each replicate. For each condition, data from all replicates were concatenated and analyzed as a single population (Figure 1 and Table 2).

**Table 2:** Statistical analysis of all sporulation/germination combinations grouped by germination condition

Condition^*a*^	Count^*b*^	Median	rCV^*c*^	Correlation^*d*^	Kruskal-Wallis test^*e*^	Mean rank^*f*^	Dunn’s test mean rank difference^*g*^	Adjusted *P*-value^*h*^
		FS log	FS log					
MM_CM	95,144	2,478.79	21.52	*r* = −0.76	*H* = 40024	459,001	17,078	<0.0001
NaCl_CM	78,242	2,486.04	21.64	*r* ^2^ *= 0.58*	*P *<* *0.0001	455,501	13,578	<0.0001
H_2_O_2__CM	96,383	2,469.32	24.06	*P = 0.0172*		450,746	8,823	<0.0001
+Fe_CM	87,260	2,469.32	22.89	—	*df* = 8	448,703	6,779	<0.0001
CM_CM	97,042	2,441.72	22.98	—	*N* = 838458	441,923	0	0
–Fe_CM	96,369	2,425.30	25.15	—	—	430,911	−11,012	<0.0001
50°C_CM	96,476	2,419.85	44.02	—	—	413,821	−28,102	<0.0001
+Cu_CM	92,402	2,350.14	27.02	—	—	402,131	−39,792	<0.0001
–Zn_CM	99,140	1,915.25	47.38	—	—	283,485	−158,438	<0.0001
NaCl_MM	97,795	1,363.85	34.91	*r* = −0.97	*H* = 73623	536,218	59,912	<0.0001
+Fe_MM	97,356	1,272.01	37.77	*r* ^2^ *= 0.95*	*P *<* *0.0001	479,074	2,768	0.1241
MM_MM	99,618	1,260.63	37.61	*P < 0.0001*	—	476,306	0	0
H_2_O_2__MM	95,216	1,249.34	40.55	—	*df* = 8	465,652	−10,655	<0.0001
CM_MM	99,510	1,232.59	43.35	—	*N* = 879122	476,306	−22,296	<0.0001
–Fe_MM	90,478	1,207.90	42.98	—	—	441,512	−34,795	<0.0001
50°C_MM	99,738	1,213.35	42.27	—	—	436,620	−39,687	<0.0001
+Cu_MM	99,607	1,162.60	47.85	—	—	416,017	−60,289	<0.0001
–Zn_MM	99,804	804.03	60.56	—	—	255,003	−221,303	<0.0001
50°C_50°C	92,197	1,000.00	44.83	*r* = −0.88	*H* = 66136	476,370	0	0
+Fe_50°C	93,361	979.97	47.46	*r* ^2^ *= 0.78*	*P *<* *0.0001	475,620	−750	>0.9999
NaCl_50°C	97,933	966.83	47.85	*P = 0.0017*	—	465,563	−10,807	<0.0001
MM_50°C	98,523	971.19	51.17	—	*df* = 8	465,089	−11,281	<0.0001
H_2_O_2__50°C	93,272	926.75	50.92	—	*N* = 850884	441,059	−35,311	<0.0001
CM_50°C	91,169	912.60	49.92	—	—	428,297	−48,074	<0.0001
–Fe_50°C	96,623	895.67	51.71	—	—	422,887	−53,483	<0.0001
+Cu_50°C	88,024	887.65	54.69	—	—	415,224	−61,146	<0.0001
–Zn_50°C	99,782	564.88	59.37	—	—	247,198	−229,173	<0.0001
NaCl_+Cu	99,651	1,266.31	36.19	*r* = −0.95	*H* = 98530	556,718	117,224	<0.0001
+Fe_+Cu	99,094	1,194.40	41.89	*r* ^2^ *= 0.90*	*P *<* *0.0001	503,498	64,005	<0.0001
MM_+Cu	99,743	1,152.19	43.20	*P = 0.0001*	—	480,441	40,948	<0.0001
CM_+Cu	99,349	1,136.75	47.76	—	*df* = 8	470,876	31,382	<0.0001
50°C_+Cu	99,816	1113.97	41.02	—	*N* = 893961	453,197	13,704	<0.0001
H_2_O_2__+Cu	99627	1,087.44	42.88	—	—	449,469	9,976	<0.0001
–Fe_+Cu	96,996	1,091.66	51.24	—	—	443,675	4,182	0.0026
+Cu_+Cu	99,783	1,,086.76	48.48	—	—	439,493	0	0
–Zn_+Cu	99,902	585.57	69.49	—	—	226,287	−213,206	<0.0001
NaCl_+Fe	99,228	1,318.61	32.21	*r* = −0.95	*H* = 134734	561,372	36,469	<0.0001
+Fe_+Fe	98,019	1,274.88	37.02	*r* ^2^ *= 0.90*	*P *<* *0.0001	524,903	0	0
H_2_O_2__+Fe	99,528	1,197.09	38.63	*P < 0.0001*	—	485,785	−39,118	<0.0001
MM_+Fe	99,685	1,183.70	42.89	—	*df* = 8	467,293	−57,611	<0.0001
CM_+Fe	99,399	1,181.04	50.89	—	*N* = 894027	461,779	−63,125	<0.0001
–Fe_+Fe	99,426	1,147.02	41.04	—	—	452,230	−72,673	<0.0001
50°C_+Fe	99,750	1,157.38	37.79	—	—	445,125	−79,779	<0.0001
+Cu_+Fe	99,206	1,134.19	46.32	—	—	442,087	−82,817	<0.0001
–Zn_+Fe	99,786	492.47	76.08	—	—	184,738	−340,165	<0.0001
+Fe_–Fe	98,514	1,154.78	39.07	*r* = −0.94	*H* = 69575	532,146	130,798	<0.0001
MM_–Fe	99,623	1,101.52	40.90	*r* ^2^ *= 0.88*	*P *<* *0.0001	496,886	95,539	<0.0001
CM_–Fe	99,585	1,079.45	42.11	*P = 0.0002*	—	486,759	85,411	<0.0001
50°C_–Fe	99,744	1,074.45	36.64	—	*df* = 8	467,520	66,172	<0.0001
+Cu_–Fe	99,288	1,041.31	42.96	—	*N* = 894698	460,698	59,350	<0.0001
H_2_O_2__–Fe	99,614	1,034.30	44.61	—	—	457,644	56,296	<0.0001
NaCl_–Fe	99,005	1,023.42	44.62	—	—	457,306	55,958	<0.0001
–Fe_–Fe	99,739	941.09	47.82	—	—	401,348	0	0
–Zn_–Fe	99,586	729.93	55.89	—	—	266,867	−134,481	<0.0001
NaCl_NaCl	96,262	926.40	46.76	*r* = −0.61	*H* = 38350	521,519	0	0
+Fe_NaCl	94,649	854.36	43.24	*r* ^2^ *= 0.37*	*P *<* *0.0001	471,595	−49,924	<0.0001
MM_NaCl	98,676	798.63	44.48	*P = 0.0810*	—	439,242	−82,277	<0.0001
+Cu_NaCl	98,386	811.30	48.34	—	*df* = 8	438,109	−83,410	<0.0001
H_2_O_2__NaCl	95,905	780.87	48.13	—	*N* = 844639	422,261	−99,258	<0.0001
CM_NaCl	98,280	734.87	52.18	—	—	399,070	−122,449	<0.0001
–Fe_NaCl	98,810	716.92	49.76	—	—	378,681	−142,838	<0.0001
50°C_NaCl	90,919	744.85	41.54	—	—	377,304	−144,215	<0.0001
–Zn_NaCl	72,752	639.24	56.88	—	—	329,666	−191,853	<0.0001
+Fe_H_2_O_2_	98,665	1,199.78	46.21	*r* = −0.79	*H* = 157131	574,968	126,910	<0.0001
CM_H_2_O_2_	99,912	1,029.66	58.63	*r* ^2^ *= 0.63*	*P *<* *0.0001	496,227	48,169	<0.0001
NaCl_H_2_O_2_	99,461	1,067.38	62.65	*P = 0.0107*	—	493,134	45,076	<0.0001
50°C_H_2_O_2_	99,781	1004.51	49.88	—	*df* = 8	488,352	40,294	<0.0001
MM_H_2_O_2_	99,818	969.01	60.11	—	*N* = 895339	476,862	28,804	<0.0001
–Fe_H_2_O_2_	99,275	920.17	66.37	—	—	453,240	5,182	<0.0001
H_2_O_2__H_2_O_2_	99,386	909.88	60.18	—	—	448,058	0	0
+Cu_H_2_O_2_	99,514	887.65	69.01	—	—	438,229	−9,830	<0.0001
–Zn_H_2_O_2_	99,527	270.17	76.08	—	—	160,729	−287,330	<0.0001
NaCl_–Zn	99,601	1,210.62	35.68	*r* = −0.99	*H* = 897081	525,981	310,313	<0.0001
MM_–Zn	99,801	1,175.74	38.20	*r* ^2^ *= 0.97*	*P *<* *0.0001	498,613	282,945	<0.0001
CM_–Zn	99,818	1,173.10	39.53	*P < 0.0001*	—	487,019	271,350	<0.0001
H_2_O_2__–Zn	99,767	1,144.79	39.64	—	*df* = 8	483,843	268,175	<0.0001
50°C_–Zn	99,821	1,152.19	40.04	—	*N* = 897081	470,437	254,769	<0.0001
+Fe_–Zn	98,771	1,108.98	39.27	—	—	456,940	241,272	<0.0001
+Cu_–Zn	99,836	1,108.98	43.28	—	—	449,778	234,109	<0.0001
–Fe_–Zn	99,757	1,099.05	40.47	—	—	449,086	233,418	<0.0001
–Zn_–Zn	99,909	667.14	60.62	—	—	215,668	0	0

a Sporulation_Germination denotes conidia transferred from solid medium sporulation environment into liquid medium germination conditions as described in Table 1.

b Number of events (cells) analyzed by flow cytometry.

c rCV = normalized standard deviation of the median, an indication of variance in the population.

d Pearson correlation analysis between median forward scatter and observed variation (rCV) within a germination group. *r* = correlation coefficient.

e The Kruskall–Wallis test determines whether there is a difference in distribution between multiple groups and is performed on ranked data. H = the Kruskall–Wallis statistic, an indication of the difference between groups; *df* = degrees of freedom. The P-values indicate the significance of differences among sporulation environments in the germination condition.

f Mean rank from Kruskal–Wallis test indicates which sporulation conditions tend to have the greatest values in the germination group.

g Dunn’s multiple comparison test. Mean rank for each sporulation environment in the same germination condition was compared to the mean rank of the same sporulation and germination conditions. Dunn’s test compares the difference in the sum of ranks between two samples with the expected average difference (based on the number of the groups and size).

h Significance: *P* > 0.05 (ns), *P*  ≤ 0.05 (*), *P*  ≤ 0.01 (**), *P*  ≤ 0.001 (***), *P*  ≤ 0.0001 (****) was determined using Dunn’s test comparing the difference in the mean ranks between each sporulation condition and matching sporulation and germination conditions.

Dormant conidia produced in all sporulation environments showed very similar forward scatter profiles except for conidia produced at 50°C, in which the forward scatter peak shifted slightly to the right, suggesting a larger size (red peaks in Figure 1). Microscopic examination showed that conidia produced at 37°C were approximately 2–3 µm in diameter, while those produced at 50°C were approximately 1.5 times larger (Supplementary Figure S2).

Not surprisingly, the rate at which conidia broke dormancy and grew varied depending on germination conditions. Conidia germinated in standard media containing sufficient metals (CM, MM) at optimal temperature (37°C) showed larger median forward scatter values indicating faster growth compared to conidia germinated in media with metal limitation (−Zn and −Fe), at elevated temperature (50°C), or subjected to stressors (+Cu, +Fe, NaCl, and H_2_O_2_) (blue peaks in Figure 1 and Supplementary Table S1). Conidia from all sporulation environments broke dormancy and grew more quickly in CM germination medium than in any other germination condition. Conidia germinated in 0.5 M NaCl (osmotic stress) generally broke dormancy and grew more slowly than those in other germination conditions. These results are consistent with previous work showing that rich medium and nonstressful conditions during germination favor more rapid dormancy breaking and growth (Schmit and Brody 1976; Meletiadis 2001; Osherov 2009).

In addition to the expected contribution of germination conditions, the rate at which conidia broke dormancy and grew varied depending on sporulation environment. The sporulation environments that favored rapid dormancy breaking and growth were not the same as the germination conditions that favored it (blue peaks in Figure 1 and Supplementary Table S2). As discussed above, 0.5 M NaCl during germination resulted in reduced dormancy breaking and growth as indicated by reduced median forward scatter (Supplementary Table S1). In contrast, osmotic stress imposed by 0.5 M NaCl during sporulation resulted in conidia that broke dormancy and grew more quickly across germination conditions as indicated by increased median forward scatter (Supplementary Table S2). In addition to NaCl medium, sporulation on MM or +Fe medium generally improved dormancy breaking and growth when compared to conidia from all other sporulation environments. Conidia from +Cu, −Fe, and −Zn sporulation environments generally performed worse when compared to conidia from the MM condition suggesting that proper metal homeostasis is necessary during sporulation as well as germination. These results show for the first time that sporulation environment impacts the ability of a medically important fungus to break dormancy and grow across multiple germination environments.

While we predicted that forward scatter peaks might shift left or right with changes in germination or sporulation conditions, we were surprised to see striking differences in the widths and shapes of peaks depending on sporulation environment (blue peaks in Figure 1). *A. fumigatus* conidia are clonal, with each conidium in a colony containing a single genetically identical nucleus produced by mitosis. Previous work has shown that conidia remain dormant until they are exposed to a carbon source and water (Osherov and May 2001), at which time individuals in the population synchronously break dormancy and start growth, with rough synchrony maintained through at least the first 12 hours (Momany and Taylor 2000). Thus, we expected that individual conidia produced in the same sporulation environment would break dormancy and grow synchronously, giving rise to relatively narrow peaks. The observed wide peaks show that genetically identical conidia within the same population break dormancy and grow at different rates. The dramatic leftward shift of post-germination peaks for sporulation conditions such as −Zn medium could be explained if Zn deficiency during sporulation killed conidia. However, viability assays with FDA and propidium iodide showed that conidia sporulated on MM and on −Zn media contained very similar, low numbers of propidium iodide stained cells and that most of the conidia that did not enlarge during germination were not dead (Supplementary Table S3).

To better understand the range of individual variation within genetically identical clonal populations of conidia, we compared the robust coefficient of variation (rCV, the normalized standard deviation of the median) for forward scatter of each sporulation/germination pair (Table 2 and Supplementary Table S2). Conidia that were produced on NaCl, +Fe, 50°C, and MM sporulation media showed lower rCV values and narrower forward scatter peaks across germination conditions, indicating less size variation among individuals in those populations. Conidia from −Zn, −Fe, CM, +Cu, and H_2_O_2_ sporulation medium showed higher rCV values and wider forward scatter peaks across germination conditions, indicating more variation in germling size (Figure 1 and Supplementary Table S2). Taken together with median forward scatter values this shows that conidia that germinate faster tend to germinate more synchronously. Indeed, there was a negative correlation between germling size (median forward scatter) and size variation (rCV) across most conditions (Table 2). The correlation between germling size and its variation was stronger when comparing sporulation environments (*r*^2^=0.9, *P* < 0.0001; Supplementary Table S2) than when comparing germination conditions (*r*^2^=0.6, *P* = 0.02; Supplementary Table S1) consistent with the idea that the environment of sporulation drives germination variation.

To determine whether the environment of sporulation has an impact on the pathogenicity of *A. fumigatus*, we took advantage of a *G. mellonella* (wax moth) infection system (Jackson *et al.* 2009). Fifteen *G. mellonella* larvae were injected with a PBS control or 10^6^  *A. fumigatus* dormant conidia produced on MM, 50°C, +Fe, NaCl, or −Zn sporulation medium. Larvae were incubated at 37°C and observed every 12 hours for 3 days. Larvae that were unresponsive in a touch test were counted as dead. Five independent replicates were performed. Most *G. mellonella* larvae injected with PBS (control) survived to the end of the experiment (88%) (Supplementary Figure S3F) and most *G. mellonella* larvae injected with conidia from any sporulation condition died by the end of the experiment (92.8%) (Supplementary Figure S3, A–E). In many replicates, larvae injected with conidia produced under conditions that led to more rapid and synchronous germination (Supplementary Table S3, MM, 50°C, +Fe, and NaCl) died more quickly than those from conditions that led to slower and less synchronous germination (−Zn) (Supplementary Figure S3, 48 hours timepoint, A–D *vs* E). However, we observed inconsistencies in host survival time between replicates likely because live *G. mellonella* larvae were shipped to the lab in separate batches and so had random differences in past exposure to stress. To separate the effects of sporulation conditions of *A. fumigatus* from random host batch effects of *G. mellonella* larvae we fit a generalized linear mixed model (GLMM) (Crawley 2012; Bates *et al.* 2020) to the data from all replicates (75 larvae from each sporulation condition for a total of 375). Even after accounting for random host batch effects of replicates, we found differences in *G. mellonella* survival times after infection with *A. fumigatus* conidia produced in the five sporulation environments (Likelihood ratio test, χ^2^ = 19.1, *df* = 4, *P* = 0.0008). The most striking difference was in average survival time of *G. mellonella* injected with conidia produced at 50°C (22 hours) compared to those produced under other conditions (30–32 hours, *P* < 0.005, Table 3).

It is not surprising that some sporulation conditions which strongly impacted the speed and synchrony of germination in sporulation/germination swap experiments did not impact virulence in *G. mellonella* infection assays. The sporulation/germination swap experiments were performed over 6 hours in uniform, defined synthetic medium (Figure 1 and Table 2) while the *G. mellonella* infection assays were performed over 72 hours in larvae that contain many different microenvironments and can deploy host defenses (Supplementary Figure S3 and Table 3). Each sporulation/germination swap experiment measured tens of thousands of germination events, while *G. mellonella* infection experiments each measured 75 events. Despite the differences in these assays, it is clear that sporulation at 50°C had a strong impact on sporulation, germination, and virulence. Compared to conidia produced under other sporulation conditions, dormant conidia produced at 50°C were larger (red peaks in Figure 1 and Supplementary Figure S2), broke dormancy and grew with moderate speed and synchrony (blue peaks in Figure 1 and Supplementary Table S2), and killed *G. mellonella* larvae faster (Supplementary Figure S3 and Table 3). We do not know the mechanism for faster killing by conidia produced at 50°C, but it might be especially important in agricultural settings since *A. fumigatus* is thermotolerant and known to grow in compost (Errasquin *et al.* 2002).

Our results show for the first time that the environment of spore production impacts the spore size, germination, and virulence of *A. fumigatus* conidia and that genetically identical conidia within a population vary in the rate of breaking dormancy and growth. That genetically identical individuals show phenotypic variation that is increased by environmental stress suggests *A. fumigatus* might employ a bet-hedging strategy to ensure survival of progeny in varied hostile environments. It seems likely that a similar bet-hedging strategy would also be used by the many fungi that produce large quantities of wind-dispersed spores across varied environments.

## Supplementary Material

jkab208_Supplementary_Data
