# Supplementary material for: Sporulation environment drives phenotypic variation in the pathogen Aspergillus fumigatus
Source: G3 (Bethesda). 2021 Jun 17;11(8):jkab208. doi: 10.1093/g3journal/jkab208 (PMC8496221; doi:10.1093/g3journal/jkab208)
Supplement: jkab208_Supplementary_Data [file jkab208_supplementary_data.zip › jkab208-suppl_data/GENETICS-G3-2021-402613-s04.docx]

**Table S1. Statistical analysis of germination conditions**

| **Germination^a^** | **Counts^b^** | **Median**  **FS log** | **rCV^c^**  **FS log** | **Corre-lation^d^** | **Kruskal-Wallis test^e^** | **Mean rank^f^** | **Dunn’s test**  **mean rank difference^g^** | **Adjusted p value^h^** |
| --- | --- | --- | --- | --- | --- | --- | --- | --- |
| _CM | 838458 | 2404 | 28.58 | r = -0.77 | H = 1578766 | 6496947 | 2135891 | <0.0001 |
| _MM | 879122 | 1213 | 45.10 | *r^2^ = 0.59* | df = 8 | 4361056 | 0 | 0 |
| _+Fe | 894027 | 1152 | 48.64 | *p = 0.0159* | *p* < 0.0001 | 4049783 | -311274 | <0.0001 |
| _-Zn | 897081 | 1106 | 43.18 |  |  | 4027523 | -333533 | <0.0001 |
| _+Cu | 893961 | 1102 | 48.76 |  | N = 7888209 | 3926138 | -434873 | <0.0001 |
| _-Fe | 894698 | 1030 | 44.98 |  |  | 3705829 | -655228 | <0.0001 |
| _50°C | 850884 | 907.8 | 53.52 |  |  | 3205174 | -1155883 | <0.0001 |
| _H2O2 | 895339 | 926.4 | 67.61 |  |  | 3171735 | -1189321 | <0.0001 |
| _NaCl | 844639 | 784.4 | 48.86 |  |  | 2610009 | -1751048 | <0.0001 |

^a^ _Germination denotes concatenated data of all conidia transferred from each of the nine solid medium sporulation environments into the designated liquid medium germination condition as described in Table 1.

^b^ Number of events (cells) analyzed by flow cytometry.

^c^ rCV = normalized standard deviation of the median, an indication of variance in the population.

^d^ Pearson correlation analysis between median forward scatter and observed variation (rCV) between germination groups. r = correlation coefficient.

^e^ The Kriskall-Wallis test determines whether there is a difference in distribution between multiple groups and is performed on ranked data. H = the Kruskall-Wallis statistic, an indication of the difference between groups; df = degrees of freedom. The p values indicate significance of differences between germination conditions.

^f^ Mean rank from Kruskal-Wallis test indicates which germination conditions tend to have the greatest values.

^g^ Dunn’s multiple comparison test. Mean rank for each germination condition was compared to the mean rank of germination in MM (the base medium for all conditions). Dunn’s test compares the difference in the sum of ranks between two samples with the expected average difference (based on the number of the groups and size).

^h^ Significance: p > 0.05 (ns), p ≤ 0.05 (*), p ≤ 0.01 (**), p ≤ 0.001 (***), p ≤ 0.0001 (****) was determined using Dunn’s test comparing the difference in the mean ranks between each germination condition and MM (the base medium).
