# Supplementary material for: Sporulation environment drives phenotypic variation in the pathogen Aspergillus fumigatus
Source: G3 (Bethesda). 2021 Jun 17;11(8):jkab208. doi: 10.1093/g3journal/jkab208 (PMC8496221; doi:10.1093/g3journal/jkab208)
Supplement: jkab208_Supplementary_Data [file jkab208_supplementary_data.zip › jkab208-suppl_data/GENETICS-G3-2021-402613-s05.docx]

**Table S2. Statistical analysis of sporulation environments**

| **Sporulation^a^** | **Counts^b^** | **Median**  **FS log** | **rCV^c^**  **FS log** | **Corre-lation^d^** | **Kruskal-Wallis test^e^** | **Mean rank^f^** | **Dunn’s test**  **mean rank difference^g^** | **Adjusted p value^h^** |
| --- | --- | --- | --- | --- | --- | --- | --- | --- |
| NaCl_ | 867178 | 1207.90 | 50.91 | r = -0.96 | H = 507181 | 4452790 | 226289 | <0.0001 |
| +Fe_ | 865689 | 1186.37 | 52.66 | *r^2^ = 0.93* | df = 8 | 4395261 | 168760 | <0.0001 |
| MM_ | 865689 | 1141.87 | 56.42 | *p = <0.0001* | *p* < 0.0001 | 4226501 | 0 | 0 |
| CM_ | 884064 | 1124.04 | 60.77 |  |  | 4115234 | -111266 | <0.0001 |
| H2O2_ | 878698 | 1108.98 | 58.51 |  | N=7888209 | 4097254 | -129247 | <0.0001 |
| 50ºC_ | 878242 | 1096.58 | 54.50 |  |  | 3991188 | -235313 | <0.0001 |
| +Cu_ | 876046 | 1074.61 | 59.74 |  |  | 3824987 | -301514 | <0.0001 |
| -Fe_ | 877473 | 1062.59 | 62.59 |  |  | 3899258 | -327243 | <0.0001 |
| -Zn_ | 870188 | 634.94 | 79.93 |  |  | 2387773 | -1838727 | <0.0001 |

^a^ Sporulation_ denotes concatenated data of all conidia from the designated solid medium sporulation environment into each of the nine liquid medium germination conditions as described in Table 1.

^b^ Number of events (cells) analyzed by flow cytometry.

^c^ rCV = normalized standard deviation of the median, an indication of variance in the population.

^d^ Pearson correlation analysis between median forward scatter and observed variation (rCV) between sporulation groups. r = correlation coefficient.

^e^ The Kriskall-Wallis test determines whether there is a difference in distribution between multiple groups and is performed on ranked data. H = Kruskall-Wallis statistic, an indication of the difference between groups; df = degrees of freedom. The p values indicate significance of differences among sporulation environments compared to MM (the base medium).

^f^ Mean rank from Kruskal-Wallis test indicates which sporulation environments tend to have the greatest values.

^g^ Dunn’s multiple comparison test. Mean rank for each sporulation environment compared to the mean rank of sporulation on MM (the base medium). Dunn’s test compares the difference in the sum of ranks between two samples with the expected average difference (based on the number of the groups and size).

^h^ Significance: p > 0.05 (ns), p ≤ 0.05 (*), p ≤ 0.01 (**), p ≤ 0.001 (***), p ≤ 0.0001 (****) was determined using Dunn’s test comparing the difference in the mean ranks between each sporulation environment and MM (the base medium).
