# Supplementary material for: Sporulation environment drives phenotypic variation in the pathogen Aspergillus fumigatus
Source: G3 (Bethesda). 2021 Jun 17;11(8):jkab208. doi: 10.1093/g3journal/jkab208 (PMC8496221; doi:10.1093/g3journal/jkab208)
Supplement: jkab208_Supplementary_Data [file jkab208_supplementary_data.zip › jkab208-suppl_data/GENETICS-G3-2021-402613-s06.docx]

**Table S3. Viability Assay^a^**

| **Sporulation Environment** | **Signal in conidia that did not enlarge (%)** | | | |
| --- | --- | --- | --- | --- |
|  | **Pre-germination (Dormant)** | **CM germination condition** | **H_2_O_2_ germination condition** | **-Zn germination condition** |
| MM sporulation | N = 43274 | N = 648 | N = 2665 | N = 2212 |
| FDA signal (Live) | 81.75 | 93.85 | 77.75 | 80.30 |
| PI signal (Dead) | 3.84 | 8.68 | 8.76 | 5.37 |
| -Zn sporulation | N = 43128 | N = 626 | N = 12525 | N = 13487 |
| FDA signal (Live) | 83.90 | 100.00 | 74.55 | 95.30 |
| PI signal (Dead) | 1.25 | 12.90 | 3.96 | 1.83 |

^a^ Conidia were produced on sporulation media, harvested and introduced to germination media for 6 h incubation exactly as described for Figure 1. Unfixed conidia and germlings were co-stained with fluorescein diacetate (FDA) and propidium iodine (PI) and fluorescence and size were analyzed immediately using flow cytometry. 20,000 events were analyzed and experiments were performed in duplicate.
