# Supplementary material for: Sporulation environment drives phenotypic variation in the pathogen Aspergillus fumigatus
Source: G3 (Bethesda). 2021 Jun 17;11(8):jkab208. doi: 10.1093/g3journal/jkab208 (PMC8496221; doi:10.1093/g3journal/jkab208)
Supplement: jkab208_Supplementary_Data [file jkab208_supplementary_data.zip › jkab208-suppl_data/GENETICS-G3-2021-402613-s07.docx]

**Supplemental Material Legends**

**Figure S1. Sporulation/Germination swap assay.** (A) Conidia isolated from a single sporulation condition on solid medium (CM, indicted by circle) were aliquoted into all germination conditions in liquid medium (indicated by flask shapes). The same process was repeated with conidia from each of the nine sporulation conditions being transferred to all nine germination conditions. Different colors represent different sporulation or germination conditions as indicated. (B) Diagram of relative conidium size and shape after sporulation, during germination, and for the first 6 h of growth. Dormant conidia are 2-3 microns in diameter. Upon exposure to carbon and water they break dormancy and begin to increase in size with swelling and germ tube emergence.

**Figure S2. Conidia produced at 50°C** **are larger.** (A) Light microscopy of conidia sporulated at 37**°**C and 50**°**C on minimal medium, 1,000X magnification. (B) Violin plot of forward scatter log scaled values of dormant (ungerminated) conidia from all sporulation environments. Dashed line represents median. Dotted line represents quantile at 25% and 75%. Kruskal-Wallis test followed by one-sided Dunn’s multiple comparison tests. Significance: p ≤ 0.0001 (****).

**Figure S3. *Galleria mellonella* survival after infection.** Fifteen *G.* *mellonella* larvae were injected with *A. fumigatus* conidia and observed every 12 h. Larvae were counted as dead if they were unresponsive to touch. Larvae were injected with *A. fumigatus* conidia produced on (A) MM, (B) 50°C, (C) +Fe, (D) NaCl, (E) -Zn, or (F) no conidia PBS control. Five independent replicates are shown. Blue: Replicate 1, Orange: Replicate 2, Grey: Replicate 3, Yellow: Replicate 4, Purple: Replicate 5. Note in panel F, Replicates 4 (yellow) and 5 (purple) for the PBS control were identical, so the yellow is not visible.

**Table S1.** Statistical analysis of germination conditions

**Table S2.** Statistical analysis of sporulation environments

**Table S3.** Viability Assay
