# Supplementary figures and images for: Sporulation environment drives phenotypic variation in the pathogen Aspergillus fumigatus
Source: G3 (Bethesda). 2021 Jun 17;11(8):jkab208. doi: 10.1093/g3journal/jkab208 (PMC8496221; doi:10.1093/g3journal/jkab208)

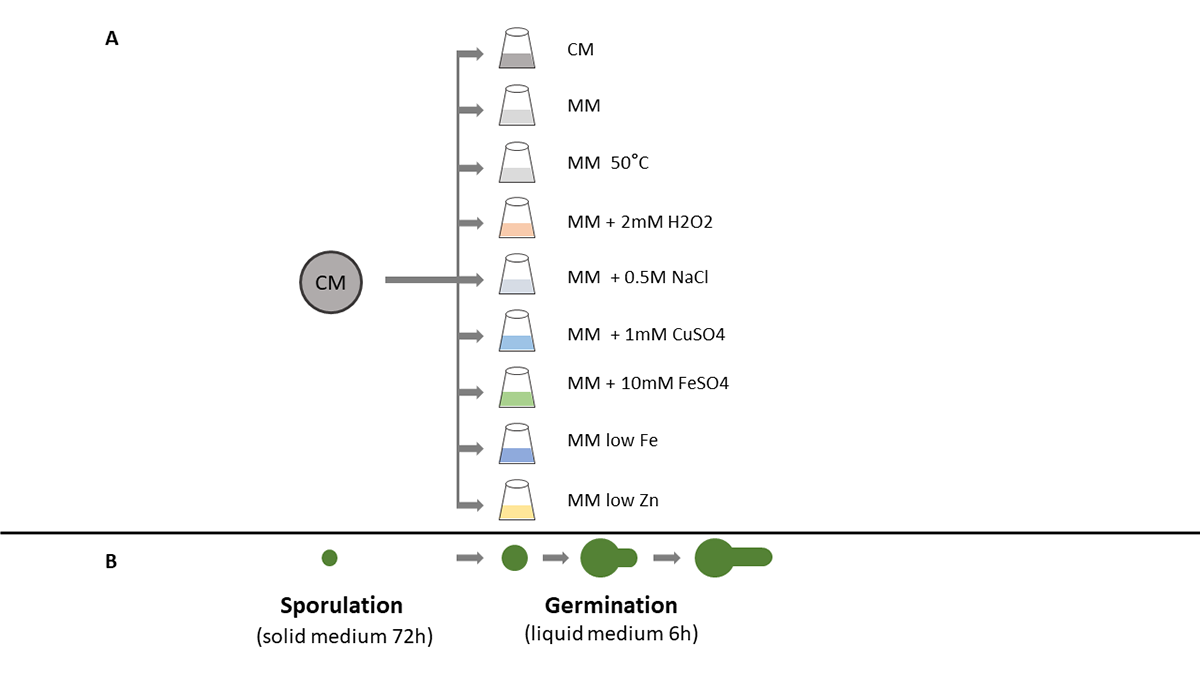

Supplement: jkab208_Supplementary_Data [file jkab208_supplementary_data.zip › jkab208-suppl_data/GENETICS-G3-2021-402613-s01.tif]

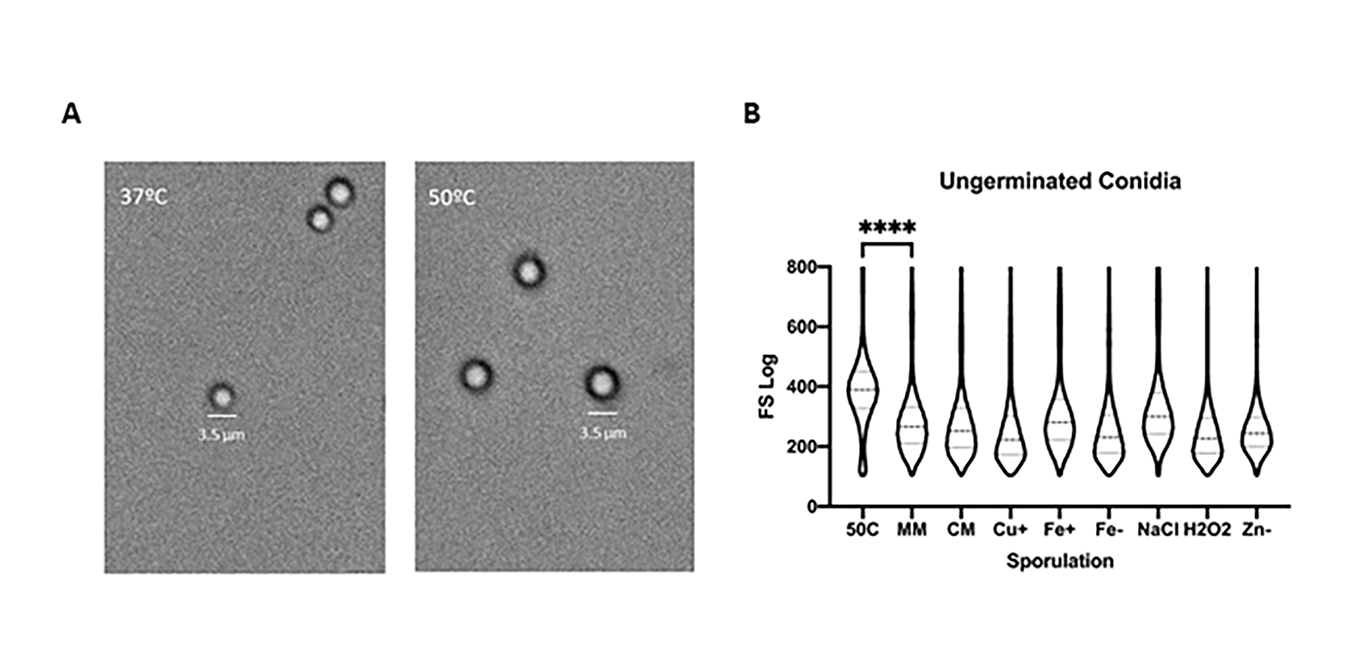

Supplement: jkab208_Supplementary_Data [file jkab208_supplementary_data.zip › jkab208-suppl_data/GENETICS-G3-2021-402613-s02.tif]

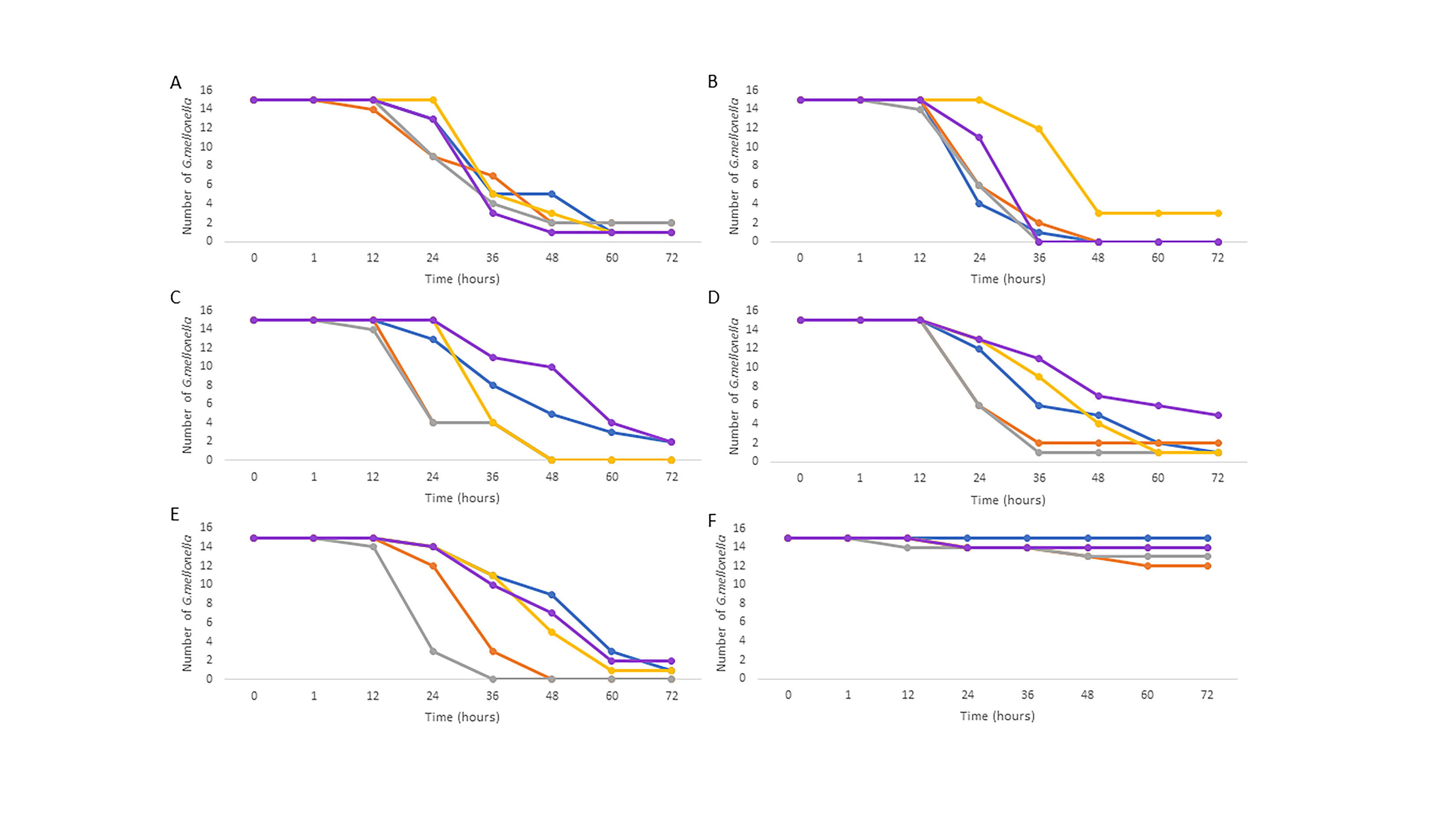

Supplement: jkab208_Supplementary_Data [file jkab208_supplementary_data.zip › jkab208-suppl_data/GENETICS-G3-2021-402613-s03.tif]
