# Supplementary material for: Sporulation environment drives phenotypic variation in the pathogen Aspergillus fumigatus
Source: G3 (Bethesda). 2021 Jun 17;11(8):jkab208. doi: 10.1093/g3journal/jkab208 (PMC8496221; doi:10.1093/g3journal/jkab208)
Supplement: jkab208_Supplementary_Data [file GENETICS-G3-2021-402613-s08.pdf › jkab208-suppl_data/GENETICS-G3-2021-402613-s08.pdf]

UNIVERSITY OF GEORGIA
Franklin College of Arts & Sciences
Department of Plant Biology

***mBio* editor and reviewer comments on original submission and two revisions**

ORIGINAL SUBMISSION

From: mbio@asmusa.org <mbio@asmusa.org>
Sent: Thursday, November 7, 2019 3:37 PM
To: Michelle Momany <mmomany@uga.edu>
Subject: mBio02743-19 Decision Letter

Re: mBio02743-19 (Sporulation environment drives phenotypic variation in the pathogen Aspergillus fumigatus)

Dear Dr. Momany:

We have completed our review of your manuscript, and I regret to inform you that we will not be able to accept it for publication in mBio. While our review indicated that the manuscript is not suitable in its current state, the work itself seems to be sound. With the necessary revisions, this manuscript could be reconsidered.

Our policy is to offer streamlined decisions and to never request extensive modifications or additional experiments; so for now, our decision must be a rejection. If in the meantime you choose to make the revisions on your own, we are willing to consider the work for one more round of review.

Thank you for your interest in mBio.

Sincerely,

Arturo Casadevall
Editor in Chief, mBio
mBioEditorInChief@asmusa.org
(Signing for the editors)

Editor comments:

As you can see from the reviewers' comments, they (and I) agree that the topic is very interesting. Both agreed in the reviews themselves and in comments to the editor that publication in mBio would require further characterization of the consequences of these differences in a relevant translational

model. Characterization of the mechanism by which variation is generated would also be an important addition to the manuscript, but mechanistic insight may be beyond the scope of this first report. You may chose to submit your work elsewhere, but we would consider a revised version (with outside reviewers) if compelling biological data were obtained that demonstrate the consequences of your interesting observation.

Reviewer #1 (Comments for the Author):

In this manuscript by Kang and Momany the impact of environmental conditions during sporulation on germination behavior of Aspergillus fumigatus is reported. The authors systematically tested nine different conditions for each spore production and germination (in total 81 combinations).
The main finding is that the environmental conditions during conidiogenesis significantly impact spore size, breaking of dormancy, germination and growth rate. Interestingly, despite their clonal origin, conidia revealed a wide range of individual variation in identically cultivated populations. The phenotypic variation is increased for conidia produced under stress conditions, implying a bet-hedging strategy of the fungus that might also be relevant for virulence.
Comments
Although this is a comprehensive study with interesting results, at the current state, I feel the manuscript remains rather descriptive as no underlying molecular mechanisms explaining the observed effects are presented. Furthermore, an obvious shortcoming of this study is that any analysis whether the described differences in germination have an impact on virulence is lacking.
In addition to flow cytometry analyses, the authors should also support their findings by providing high resolution microscopy (e.g., SEM, AFM) of conidia. Are there any obvious differences of the spore surface? Transmission electron microscopy could provide data on whether the thickness of the conidial cell wall is affected.

Reviewer #2 (Comments for the Author):

The authors report a very interesting study on factors that affect germination efficiency in the human pathogen, Aspergillus fumigatus. Although it is well-known that nutrient and stress conditions during germination are major drivers of germination rates, the impact of sporulation conditions are relatively unknown. Heterogeneity amongst isolates is known to impact virulence in Aspergilli and the rate of breaking dormancy is potentially one such attribute. This study attempts to directly address this question in a single genetic background. The authors provide data to show that sporulation of Aspergillus under varying environments can impact the rate at which the population of conidia breaks dormancy, inducing heterogeneity independent of genetics. The author propose that these findings suggest a bet-hedging strategy wherein Aspergillus (and potentially other conidia/spore-based pathogens) respond to environmental stress by increasing variation in phenotypes to ensure survival of

clonal progeny. Overall, this is a short but interesting study. I have only a few comments for the authors to consider.

1. Although very interesting, the study needs a translational component. If sporulation under different conditions drives variability in germination, and that variability ensures survival in the hostile host environment, then populations of conidia with increased variability should display altered virulence phenotypes in mouse models of invasive aspergillosis (or, in vitro cell interaction assays). Are conidial populations with more synchronous, faster germination rates more/less "virulent" than those with more variable, slower rates? The studies currently available in the literature utilize genetically unrelated strains to test this, whereas this study could directly address this question in a manner independent of genetic background.

2. How did the authors control for conidial clumping during the germination experimets? Germinating Aspergillus conidia, especially in shaking broth culture, are prone to forming clumps due to cell wall changes. While cell wall changes are typically temporally coupled to other germination events, it is possible that the varying sporulation/germination conditions could have uncoupled these properties and altered this phenomenon independent of breaking of dormancy.

REVISION 1

From: mbio@asmusa.org <mbio@asmusa.org>
Sent: Thursday, June 25, 2020 2:55 PM
To: Michelle Momany <mmomany@uga.edu>
Subject: mBio01461-20 Decision Letter
June 25, 2020

Re: mBio01461-20 (Sporulation environment drives phenotypic variation in the pathogen Aspergillus fumigatus)

Editor comments:

The report remains novel and interesting and the Galleria data have the potential to provide a demonstration that the differences in germination are biologically meaningful. The new data do not include a statistical analysis and thus do not convince the reader that the differences are significant. Perhaps with the support of a statistician, an analysis of the differences of killing must be included. If a meaningful significant difference that corresponds with the germination analysis is observed that correlates with a difference in gemination, a revised manuscript can be considered.

UNIVERSITY OF GEORGIA
Franklin College of Arts & Sciences
Department of Plant Biology

REVISION 2

From: mbio@asmusa.org <mbio@asmusa.org>
Sent: Friday, April 16, 2021 10:52 AM
To: Michelle Momany <mmomany@uga.edu>
Cc: sekang2@gmail.com; Brandi Nicole Celia <Brandi.Celia@uga.edu>; Douda Bensasson <dbensasson@uga.edu>
Subject: mBio00856-21 Decision Letter

Re: mBio00856-21 (Sporulation environment drives phenotypic variation in the pathogen Aspergillus fumigatus)

Editor comments:

The manuscript by Monomy et al. addresses the question of whether spores formed under different conditions have different characteristics. In order to do so, the authors sporulated Aspergillus fumigatus under different conditions, then germinated the spores from each condition in the nine different conditions. For both sporulation and germination, nine conditions were used including rich and minimal medium complete medium, minimal medium, high temperature, excess copper or iron, limited iron or zinc, or high salt or ROS. Flow cytometry was used to quantify germination. One of the more conclusive findings was that spores formed at 50 degrees were larger and associated with decreased virulence. *[Momany comment: this is backwards. 50C spores are more virulent.]* Other conditions led to more subtle differences in the germination rate and synchrony. *[Momany comment: some of the other conditions showed large differences in germination rate and synchrony, but the differences in pathogenicity were more subtle,]*. The work, while supported by robust statistical analyses, includes no efforts to mechanistically explain the differences across conditions. The authors addressed the previous critiques, but did not advance the work beyond a descriptive characterization. The work will be a useful resource for investigators to indicate that the conditions under which sporulation occurs may have biological consequences.
